# Designing digital nature for older adults: A mixed method approach

**DOI:** 10.1177/20552076231218504

**Published:** 2023-12-03

**Authors:** Josca van Houwelingen-Snippe, Somaya Ben Allouch, Thomas J. L. van Rompay

**Affiliations:** 1 Department of Communication Science, Faculty of Behavioural, Management and Social Sciences, 3230 University of Twente, Enschede, The Netherlands; 2 Digital Life, 10191 Amsterdam University of Applied Sciences, Amsterdam, The Netherlands; 3 Digital Interactions, Informatics Institute, 1234 University of Amsterdam, Amsterdam, The Netherlands

**Keywords:** Older adults, digital nature, digital interactions, social well-being, social connectedness

## Abstract

**Objective:**

Loneliness and social isolation are pressing issues that can seriously impact the mental health and well-being of older adults. Interacting with nature can stimulate a feeling of connectedness. However, for older adults, access to nature is often troublesome because of physical limitations and mobility restrictions.

**Methods:**

In the present mixed-method study, 37 older adults (62–99 years old) with varying care needs and mobility restrictions watched a video presenting a walkthrough of a simulated digital nature landscape.

**Results:**

Quantitative results show a significant increase in social connectedness scores and enhanced peacefulness after experiencing a digital nature. Qualitative results stress the importance of variations in nature scenery and highlight the influence of contextual and person-related factors including nature experiences throughout the life span and mobility constraints that older adults may face.

**Conclusion:**

These findings testify to the potential of using digital nature as a complementary strategy when interactions with outdoor nature become increasingly difficult due to old age.

## Introduction

Loneliness and social isolation among older adults pose serious public health concerns, compromising both physical and mental dimensions of well-being.^1–3^ Interacting with nature can reduce feelings of loneliness, boost connectedness, and enhance feelings of social support.^4–6^ These findings indicate that when people feel more related to nature, they feel more connected to the world at large.^7^ However, for older adults, real-life nature interaction is often troublesome because of physical vulnerabilities (e.g., fear of falling), related mobility constraints^8,9^ or because nature is simply not around in urbanized regions. As a result, those who need nature interaction the most are often also the ones for whom access to nature is troublesome.

However, a growing body of literature shows that providing opportunities for nature interaction indoors could provide an alternative means to safeguard contact with nature, especially when considering that indirect contact with nature, such as watching nature pictures or videos, can have similar effects on well-being as real-life nature interaction.^10–12^ To illustrate, being exposed to a screensaver presenting an awe-evoking nature scene for a few short moments a day can boost mood and trigger positive emotions.^13,14^ This latter finding aligns with recent studies using immersive digital nature which show that awe-evoking and vast digital nature in particular stimulates prosocial behavior and feelings of connectedness.^15–17^

These findings hint at the potential of immersive technologies (such as virtual and augmented reality) as they are associated with high levels of immersion, defined as the sense of being present in the simulated environment.^18^ In addition, they provide the opportunity to design environments embodying specific nature characteristics, including *fascination* and *spaciousness*.^19^ Hence, when real-life nature interaction is compromised due to old age, digital nature (including all simulated 3D nature graphics displayed using various forms of technology, ranging from projectors to head-mounted displays) provides an alternative means to interact with nature.^13,20–22^ In addition, digital nature might help people reconnect with outdoor nature or augment human–nature interactions.^23^

To further explore the impact of digital nature, a study was conducted in which older adults were exposed to digital nature designed based on research findings stressing the importance of spaciousness, safety, and comfort perceptions.^24^ Using both quantitative and qualitative research methods, the present undertaking not only aims at pinpointing essential nature characteristics but also aims at identifying person-related and contextual variables that influence digital nature experience so as to provide initial guidelines for the implementation of digital nature in the lives of older adults.

### Digital nature and its potential for well-being

In recent years, findings from diverse studies highlight the potential of immersive nature simulations, either making use of head-mounted displays, projections in lab or care settings, or computer displays in people's home environments.^25,26^ For instance, a recent study conducted during the recent COVID-19 lockdowns showed that even a 4-min digital walk through nature made participants feel more connected to their community.^27^ Additionally, creative concepts including virtual windows^28^ and biking exercises with augmented nature^29–31^ underscore the potential of digital nature to allow enjoyment of nature's benefits regardless of age and physical impairments.

Although the potential of digital nature as a complimentary means of nature interaction is increasingly recognized, insights into interaction types and related environmental characteristics particularly well suited to enhance social and mental dimensions of well-being (including connectedness and related emotions) are limited, subsequently thwarting attempts at formulating evidence-based design guidelines for simulated nature environments.

#### Nature interaction; spaciousness and feeling safe

Across disciplines including emotion research, nature studies and healing environments research, the importance of spaciousness has been pointed out. For instance, interpersonal connectedness and self-disclosure are greater in spacious rather than confining indoor settings,^32^ creativity and positive affect are boosted when people are exposed to wide-open, rather than dense, park and forest settings,^12^ and vast nature settings are associated with the experience of awe^33^; an emotion which brings along a high level of perceived connectedness with others and the world at large, and that stimulates prosocial behavior.^19,34^ For instance, a spacious nature setting resembling an open field was associated with higher levels of social aspirations compared to a dense digital nature setting.^35^ However, a follow-up study with older adults could not replicate these findings,^24^ arguably because spacious settings lack the more intimate, secure character associated with more secluded nature settings.

As a matter of fact, when it comes to nature preferences for older adults, safety concerns indeed play a particularly important role.^36^ For example, logistic landscape characteristics such as well-maintained paths and facilities to sit down become increasingly important with old age.^36^ Additionally, in an online survey, independent adults aged over 55 years old experienced more social aspirations when being exposed to a tended nature scene (including the presence of benches, well-maintained walking paths and other cues signaling human presence) compared to a wild nature scene.^24^ Hence, rather than being attuned to opportunities for excitement and exploration (that are more readily associated with wild and vast nature^37^, for an older population, concerns for safety and comfort might prevail due to aforementioned mobility restrictions and related reductions in autonomy.

In line with the aforementioned literature showing that older adults value logistic nature characteristics, such as well-maintained paths and places to sit down^36^ and tended rather than wild nature,^24^ arguably social aspiration and emotion scores are higher for participants who experienced a simulated walk through a tended, rather than wild, nature scene. As for the overall effect of nature interaction on feeling connected, we hypothesized an increase in feeling connected (pre- vs postexposure) for older adults in the present study. Finally, in light of the inconclusive findings regarding the effects of spacious (versus more dense) scenery on the social and mental well-being of older adults, participants either experienced a nature scene that gradually becomes more spacious or one that becomes more denser.

To address these predictions and to further explore the effects of spaciousness on well-being measures (open research question), we used a mixed-method approach; the first (quantitative) part of the study employed an experimental design, whereas the second (qualitative) part involved semistructured interviews to gain deeper insights in participants’ experiences of the digital walk and contextual factors involved.

## Method

### Stimulus development

To arrive at the four videos for the 2 (spaciousness: *dense* vs *spacious*) × 2 (type of nature: *wild* vs *tended*) between-subjects design employed in this study, four different simulated nature walks with identical auditory stimuli (soundtrack with bird sounds and footsteps) were developed using advanced game development software (Unity; Gaia Package; https://assetstore.unity.com/packages/tools/terrain/gaia-terrain-scene-generator-42618). All four videos^1^ consist of a 30 s stationary viewpoint for the first part, after which the viewpoint turns around (180° turn) and follows a path through a virtual forest scene for 220 s to the final destination where the viewpoint remains stationary for another 30 s. The virtual environment further presents the movement of clouds, trees, vegetation, and corresponding shadows.

**Figure 1.**
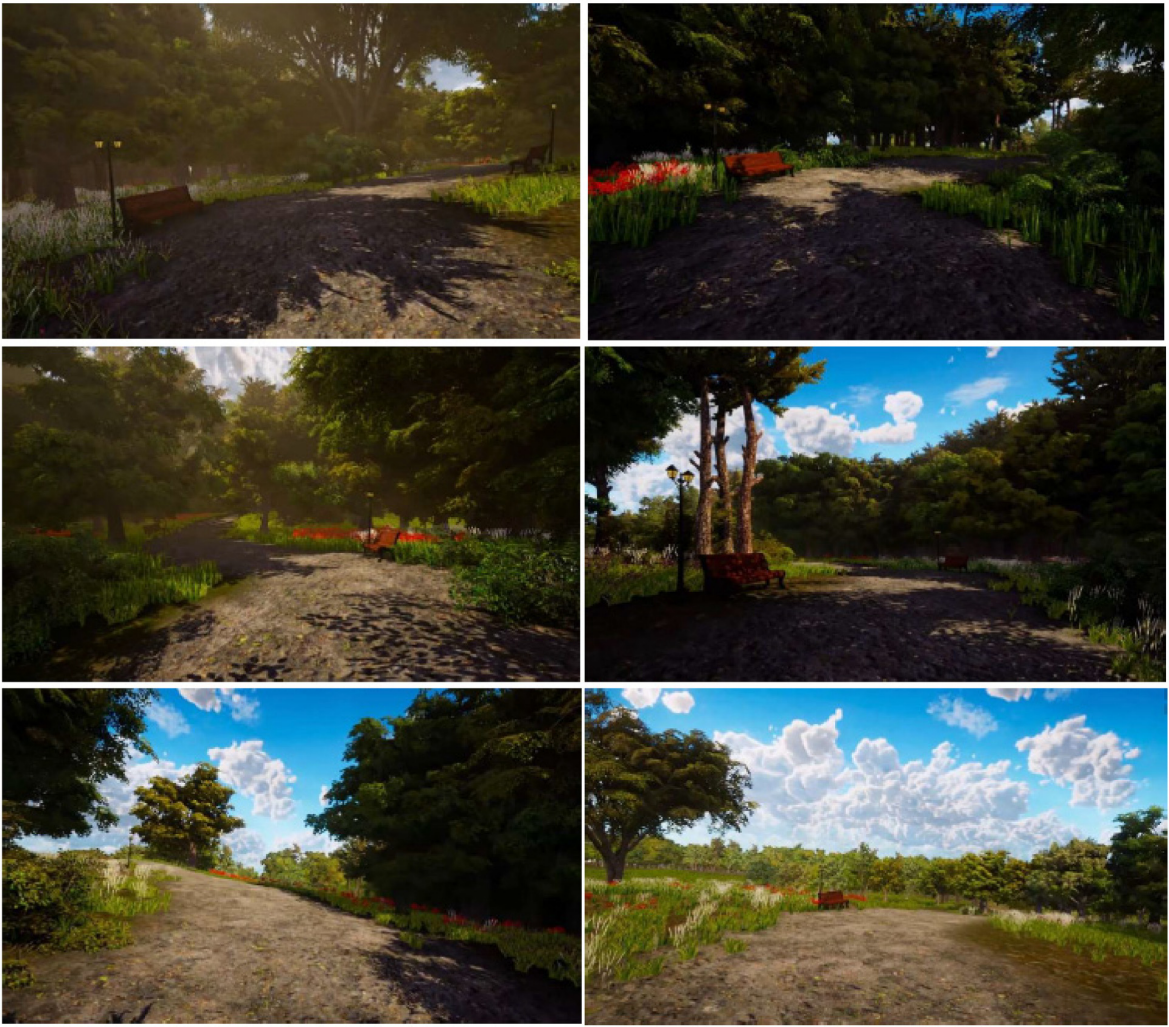
Screenshots of the trajectory shown in the spacious (transition from dense to spacious scenery) and tended (including flowers and benches) digital nature condition.

The experience generates the feeling of a walkthrough by presenting movement, accompanied by the sound of footsteps and a gradual change in scenery. The walkthrough either starts at a dense part of the forest and gradually turns into a spacious scene, or vice versa (spaciousness manipulation). In addition, the conditions either display a tended nature scene, with flowers and benches, or a wild nature scene, without any human intervention (type of nature manipulation). Figure 1 shows various screenshots throughout the trajectory.

### Participants

Older adults (*N* = 37, age range from 62 to 99 years old) who received care from two Dutch care organizations participated in the study. Participants were recruited by care professionals at in total seven different locations of the respective care organizations. Care professionals considered whether inhabitants were able (i.e., to express their opinions and to provide informed consent) and would enjoy participating. In total, 38 older adults were recruited for the study, however, one participant opted to withdraw from the study before watching the video.

### Procedure

In March 2021, the first series of research appointments were planned. The research appointments were either planned in a designated room at the care center or at the participant's home. Due to COVID-19 restrictions, only care professionals were physically present, whereas the researcher responsible for data collection joined online via a video conference application (Microsoft Teams). When the lockdown restrictions became less stringent (July/August 2021), the researcher was allowed to join, while still maintaining social distancing norms.

After explaining the goal of the study, participants filled out the informed consent form and pretest measures for connectedness to the community and (positive and negative) emotions. Subsequently, participants watched one of the four (randomly assigned) digital nature videos (each participant watched the simulated nature walk on the same laptop in full-screen mode with sound on). Next, participants filled out the remainder of the questionnaire comprising the posttest measures and additional outcome measures.

Subsequently, the (semistructured) interview was conducted. During the first part, the participants were asked to reflect on their experience and opinion of the digital walk through nature they experienced. During the second part, participants were asked to elaborate on the (social) context in which they experience(d) nature in daily life and under what circumstances participants would appreciate and turn to digital nature if it would be available at the care center. The procedure was approved by the ethical committee of the University of Twente (request number 200145).

### Measures

A 5-point Likert scale was used for all measures.

#### Connectedness to the community

**Figure 2.**
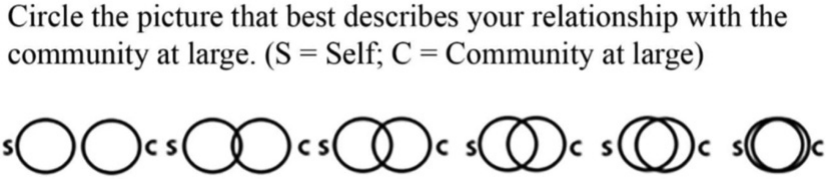
The inclusion of community in the self (ICS) scale.^38^

Connectedness to the community was measured using the Inclusion of Community in the Self Scale.^38^ This single-item measure was used as a *pre*–*post* test measure; see Figure 2.

#### Positive and negative emotions

To measure participants’ emotions, items derived from the modified Differential Emotions Scale^39^ (were used. Of the original scale with 10 positive and 10 negative emotions, three positive emotions (high on valence and low on arousal) and three negative emotions (low on valence and high on arousal) were used. These emotions were deemed most appropriate here considering their relatedness to stress reduction and positively affect induction^40^; two key benefits of natural interaction:

1. To what extent do you feel angry, irritated, or annoyed at this moment?
2. To what extent do you feel stressed, nervous, or overwhelmed at this moment?
3. To what extent do you feel sad, downhearted, or unhappy at this moment?
4. To what extent do you feel serene, content, and peaceful at this moment?
5. To what extent do you feel interested, alert, or curious at this moment?
6. To what extent do you feel inspired, uplifted, or elevated at this moment?

#### Social aspirations

Social aspirations were measured with the Social Aspirations scale^35^ with items tapping the appropriateness of the nature scene for social interaction as indicated by items such as “*I would like to show this landscape to someone*” and “*This landscape is suitable to experience together*.” Due to poor internal consistency, the only reversed item was excluded, resulting in a four-item scale (α = .62).

#### Awe (connectedness)

Connectedness was measured with the connectedness subscale of the Awe Experience Scale^41^ which includes items such as “*I experienced a sense of oneness with all things*,” “*I felt a sense of communion with all living things*,” and “*I felt closely connected to humanity*” (α = .77).

#### Sense of presence

To investigate the effects of the different conditions on immersion (e.g., sense of presence), three items of the self-location construct of the original “Spatial Presence Experience Scale”^42^ were used that tap into the extent to which one feels more or less present in the environment of the video (e.g., “*I felt like I was actually there in the environment of the video*”; α = .70).

## Analysis

### Quantitative analysis

The analysis of the quantitative results included a pre–post test of the two pre–post measures, that is, the connectedness measure and the positive and negative emotions. Furthermore, the original plan was to run multiple regression analyses to predict Social Aspiration, Awe (Connectedness) and Sense of Presence scores from the experimental conditions Spaciousness, Type of Nature and Spaciousness × Type of Nature. However, since not all assumptions of the analysis were met (the data were not normally distributed), and the test was clearly underpowered, only descriptive statistics are presented.

### Qualitative methodology and analysis

Common themes and concepts were identified by carefully analyzing all transcripts. The coding process used a mixed-method approach, such that some of our themes were based on prior knowledge from literature (deductive approach) while other themes emerged directly from the participants’ narratives (inductive approach).^43^ The authors performed several rounds of coding to compare new codes to previously assigned codes, to make sure the identified themes remained valid and to derive the final set of themes.

The coding process was performed using Atlas.ti 9 software and consisted of three stages, derived from^44^: (1) open coding, in which the themes emerging from the transcripts were coded in vivo using constant comparison, (2) axial coding, where themes were connected and combined into categories, and (3) selective coding, in which a storyline was uncovered based on the recurring themes.

The coding process was performed by the interviewer, with constant collaboration with the other authors. Ten percent of the transcripts were coded by a second coder and the interrater agreement was almost perfect (*κ *> 0.9).

## Results

### Demographics

The demographics of the study population are presented in Table 1. The participant selection aimed to reach older individuals with varying care needs. The only requirement for participation was that participants were still able to give informed consent themselves, and thus were able to consciously make this choice. While the sample size is limited, it is representative of older adults in Dutch care centers.

**Table 1.** Participant characteristics*.*

Category	Specification	Number of participants
Year of birth	<1930	4
	1930–1934	9
	1935–1939	9
	1940–1944	7
	1945–1949	4
	>1950	4
Gender	Male	13
	Female	24
Marital status	Married	12
	Never married	5
	Divorced or separated	3
	Widowed	17
Living situation	Alone	30
	Together with others	7
Care level	Nursing home	17
	Assisted living	14
	Home care	6
Education level	Low	16
	Middle	14
	High	7
Ability to go outside alone	Yes (no physical limitations)	8
	Yes (with electronic wheelchair)	7
	Limited	9
	No	13
Living area	Rural	15
	Urban	22
Number of physical nature interactions during a week	Less than once	15
	1–2 times	12
	3–5 times	4
	More than 5 times	6
Outdoor area	Garden	5
	Balcony	14
	Garden and balcony	3
	None	16

### Connectedness (pre- vs post-exposure)

Before and after experiencing the digital nature video, participants answered the single-item connectedness to the “community at large” measure. A Wilcoxon signed-rank test indicated that postexposure connectedness scores were significantly higher (*M *= 4.59, *SD *= 1.09) than preexposure scores (*M *= 4.22, *SD *= 1.21; *Z *= −2.652, *p *< 01), confirming that feelings of connectedness are higher after watching digital nature.

### Positive and negative emotions (pre- vs postexposure)

A Wilcoxon signed-rank test indicated that postexposure ratings for *serene, content, and peaceful* were significantly higher (*M *= 3.94, *SD *= 0.58) than preexposure ratings (*M *= 3.47, *SD *= 0.83; *Z *= −2.995, *p *< .01). Additionally, a Wilcoxon signed-rank test indicated that postexposure ratings for *angry, irritated, or annoyed* were marginally lower (*M *= 1.06, *SD *= 0.23) than preexposure ratings (*M *= 1.21, *SD *= 0.58; *Z *= −1.890, *p *= .06). The Wilcoxon signed-rank test showed no significant differences in pre- versus postexposure ratings for the other emotions items (*p*s > .10).

#### Descriptive statistics

As discussed, the sample size was too limited to test for differences across the four different nature conditions (and even when ignoring this limitation, regression analyses showed no significant effects; all *p*s > .35) For reasons of completeness, for each of the outcome measures (i.e., social aspirations, connectedness, and sense of presence) descriptive statistics are presented in Table 2.

**Table 2.** Descriptive statistics for social aspiration, awe (connectedness) and sense of presence.

Variable	N	*M*	*SD*
**Experimental conditions**			
Social aspirations	36	4.229	0.529
Awe (connectedness)	36	3.839	0.664
Sense of presence	36	3.713	0.933

## Conclusion

The findings presented for the pre/posttest measures indicate that participants in the present study felt more connected after (rather than before) experiencing digital nature and felt more *serene, content, and peaceful* and marginally less *angry*, *irritated,* and *annoyed*. However, we could not demonstrate significant differences across our different nature conditions. To further explore participant experiences and to gain insights into person-related and contextual influences affecting digital nature experience, semistructured interviews were conducted.

### Aim of semistructured interviews

After the collection of the quantitative data was completed, the semistructured interview started which consisted of three parts: (1) investigating the emotional responses and preferences concerning the digital nature videos in general, (2) the experimental manipulations specifically, and (3) to explore preferences for additional digital nature content.

### Qualitative results

Within this section, the qualitative results are presented in the following order: Emotional responses and preferences concerning the digital nature videos, improvements, and suggestions for additional digital nature content.

### Emotional responses and preferences concerning the digital nature videos

All participants experienced a 4-min video, see section Stimulus Development. Subsequently, the participants were asked to describe the experience of the video and to name specific elements in the video they enjoyed and specific elements that need improvement. Within this section, first the experiences of (and responses to) the digital nature videos are discussed. Subsequently, the most frequently mentioned suggestions for improvements are presented.

### Positive experiences with the videos

The vast majority of the participants of this study expressed that the experience of the video was enjoyable and a positive experience in general. Some participants elaborated precisely what specific elements of the video were perceived as enjoyable. In the following fragment, for example, participant 13 discussed the emotional effect of watching the video had on her (describing the spacious scene portraying an isolated tree which made her feel strong):

*Participant 13: I am crazy about trees and what I thought was very beautiful, at one point at the very beginning, there was one tree and it stood so powerful in nature, well rooted, so that is how I felt at that moment as well.*

This fragment also illustrates the importance of landmarks or specific elements in the digital nature scene that direct attention and allow for perspective-taking.

Immersion as a theme was covered in the survey, however, it also came across during the interview, such as in the following fragment where Participant 13 points out that she had a sense of presence in the environment of the video. She describes the experience of the video as if she was actually there:

*Participant 13: Well, I was there in an environment that I really liked, because I like trees and I like greenery. And so I found myself completely in the green and I loved it there, I completely lost track of where I actually was.*

The scenery was generally inviting as a whole, according to Participant 31. She even expressed the wish to visit the scene in real life.

*Participant 31: I saw a country road and those flowers on both sides, the trees, different types of trees, a beautiful sky and the clouds, yes, and that ascending road, was really inviting. […] Yes, inviting, that's what it actually looked like in its entirety. […] I love to find such roads, such paths and to be a bit in thought and look around, yes, wonderful.*

### Emotional responses and preferences concerning spacious characteristics

The videos displayed a transition with a contrast experience, which for some participants resulted in emotional responses which is illustrated clearly by the following fragment where participant 19 described her experience while transitioning from a more open to a more dense section of the scene.

*Participant 19: Yes, that transition, that oppresses at first, doesn't it? At first it's a bit oppressive and then you come to rest and then you think: yes, that's a nice area here. But you have to get through that tightness first.*

In the following fragment, participant 27 elaborated on the emotional response to the transition from a dense to a spacious scene. This transition was described as a positive surprise.

*Participant 27: At a certain point, especially between those trees, I thought that was very nice, then you arrive at that wide part all of a sudden. Yes, I think that's very nice*.

When Participant 27 subsequently was asked to elaborate on what she enjoys in a more open space, she highlights that being able to see far ahead and watch the sky is important for her.

*Participant 27: The fact that you can see far and I often enjoy the skies. Especially the beautiful Dutch skies with those beautiful clouds in them. […] I think that's wonderful, I think that's very beautiful.*

There were also participants who preferred the dense part of the video over the more spacious part. For example, the following participant who especially described that in her perception the colors became pale and the scene overall less enjoyable when going from the dense to the more spacious scene.

*Participant 22: Yes, I started more in the middle of the forest and then I came out in the middle of the day or something. I noticed that difference. […] I liked the first part the most. The most recognizable for me. […] And the last part came across as more faded to me.*

The preference for either the more dense or the more spacious part of the video seems to differ across individuals. As suggested by the previous passage (Participant 22), this preference may be determined by *familiarity* or *recognizability* of the scene presented.

### Emotional responses and preferences concerning tended nature elements

In the description of the environment in the video, many participants in the tended nature conditions described the benches as a nice opportunity to rest. Often this is related to mobility issues participants experience in real life. In the following fragment, participant 34 described the desire to sit down on one of the benches in the video and to enjoy nature, which came up frequently in the interviews.

*Participant 34: […] and that red bench appeared again and again. I thought maybe, yes there you can, well, sit down and watch nature quietly, at your leisure.*

Some participants also brought up the absence of people in the environment in the video with regard to the benches:

*Participant 17: […] and often those beautiful benches and then I kept thinking ‘Why is no one sitting there, on such a beautiful bench?’ […] I'm not walking well, and then I see a bench like that where I can sit nicely on, rest for a while.*

### Responses to the absence of tended nature elements

Interestingly, a number of participants in the wild nature condition described a more tended scene when they were asked what they would want to see differently in the digital environment they had just experienced.

*Participant 31: You can put a bench in it or show people in the environment or someone walking their dog. […] I would like to see a bench in it, because if you are alone you can sit there for a while, but if you are with someone, you can also sit down for a while.*

In the following fragment, the absence of people in the video comes up again. Participant 29 argued that the environment in the video of the wild nature condition was desolate, stating that elements indicating human interference or human affordances (i.e., elements in a tended nature scene) would already be an improvement:

*Interviewer: But would it be an addition for you if we indeed show people in the video, or would you like to see something else?*

*Participant 29: Well, that some people are walking, people with a dog or a couple with a pram? Something like that. I thought this was gloomy, of course, because there was nothing else*.

*Interviewer: We also have videos with benches and lanterns, so it gives a bit more of a park feel. Could that also be a way?*

*Participant 29: Yes, yes, it certainly could.*

In general, there seemed to be a preference for tended nature scenes within this study.

### Improvements regarding the digital nature video

Although participants were motivated to speak freely, when asked for possible improvements regarding the video participants had just watched, most participants could not come up with anything to change. The most frequently mentioned improvements for the video mentioned by participants (discussed next) were related to the sound of the footsteps in the video and the desire to add liveliness to the scene in the form of human presence or the addition of animals.

#### Sound of footsteps

As an improvement, the sound of the footsteps came up several times, especially because participants could not match the sound with what they saw, as the following fragment illustrates:

*Participant 11: But the footsteps were very disturbing. […] Those footsteps sound like they are on concrete and you are walking a forest path, on a dirt path. So I find that very disturbing, otherwise I think the video itself is well made.*

#### Means to add liveliness to the digital nature scenes

Participants often expressed the desire to add some liveliness to the digital nature scene. A negative remark some participants made in regard to the video was that they perceived the scene to be desolate, empty or monotonous or even that watching it made them feel lonely. In the following fragment, the absence of people comes up.

*Participant 29: When you saw that video, it was trees, a path, etcetera, etcetera, and if you don't see anyone else, it's very lonely. It would make you sad if you watch that and no one is walking there.*

Participant 29 subsequently pointed out that from his window, he could see more activity in the streets, suggesting that the mere presence of other people in the scene would be an improvement.

Another possible improvement as a way to enhance the liveliness of the scene that was frequently suggested (by more than half of the participants in the present study) concerned the addition of animals in the video. It gives you something to look at, some liveliness and activity to watch. Some of the participants also referred to the fact that the video contained the sound of birds without having visual birds flying around.

*Participant 25: Yes, beautiful nature! Beautiful flowers, beautiful trees, yes, grass, benches with beautiful lanterns. Only no animals, I missed that, the animals, the deer, the bunnies.*

To sum up, most participants described the experience of the digital nature scene as overall positive. While there were individual differences in the preferences for spaciousness, tended nature elements seemed to be associated with the possibility to sit down and enjoy nature. Even participants who experienced digital nature scenes without the tended nature elements (i.e., participants exposed to the wild nature condition), expressed the desire for tended nature elements suggestive of human presence.

In terms of improvements, the sound of the footsteps created a mismatch with the visuals for some participants. Some participants considered the digital nature scene monotonous or desolate, likewise suggesting the addition of tended nature elements or human presence in the scene. A large number of participants described past nature interactions featuring animals and explained their preference by stating that it would give the user something to watch.

### Suggestions for additional digital nature content

In the second part of the interview, the concept of a projection room with digital nature projections within the care center where the participant was living was introduced. Participants were subsequently asked whether they would like to experience different digital nature content every time they would use the projection room, and if this was the case, what different scenes participants would prefer.

### Variety in scenery

During this part of the interview, the participants were asked what kind of scenes they would want to experience if they had the opportunity to experience nature projections repeatedly on a day-to-day basis. Almost all participants indicated that they would like to see different projections, pointing out that seeing the same scene over and over again is boring:

*Participant 16: When I've seen something, I long for something else. Well and I think that's tricky too, if I've seen it twice, I'll be bored.*

*Participant 4: There may of course be a video that you then see again, that is not so bad. But it's nice to have a little variety.*

When participants were subsequently asked to describe what kind of scenes they would like to see, and if the scenes should be different every time, the answers varied considerably, illustrating the heterogeneity of older adults as a target audience. Participant 32 explained that a specific scene could possibly be interesting for the duration of a month, but after that she would want to see different scenes from different locations:

*Participant 32: Yes, but you know, that's nice for say a month. But after that month I would also like to see farmlands and cows, for example. Or a beautiful snow landscape in the Alps and such, I think that's beautiful too. […] A landscape, which I also thought was very beautiful, was the desert.*

Other participants indicated that Dutch scenes would be sufficient, for instance explaining that nature in the Netherlands is varied:

*Participant 38: You have beautiful nature everywhere, the Netherlands is quite beautiful. […] But [nature in] the Netherlands is varied.*

Participant 21 shared this point of view, she also expressed no need for scenes from all over the world.

*Participant 21: Oh no, I don't need to see scenes from the other side of the world. Nature is also beautiful here.*

Participant 34 indicated that what she would like to see is dependent on her mood. In line with the importance of compatibility between scene and mood,^37^ Participant 34 indicated that what she would like to see is dependent on her mood:

*Participant 34: One time, you think, oh wonderful, enjoy the sun, and everything quiet and peaceful. And the other time, you also want to kick something because you are angry, or feel bad about yourself.*

#### Desire for vastness and blue space

When it came down to what type of scenes participants would enjoy, scenes with blue space and vastness in general were mentioned, in particular the sea or water areas in general were recurring themes. These blue spaces provide a spacious scene with an overview. Participant 26 pointed out the attractiveness of a wide view over the sea as it provides a lot to watch:

*Participant 26: Terrace by the sea. That may well be in the Netherlands. [You can watch] the people, and all those vistas; above the sea, ships maybe in the distance, if you’re lucky.*

Participant 10 also mentioned the sea, especially for the peacefulness of the scene.

*Participant 10: I love the sea. […] It changes with the moment and you find peacefulness there.*

Other participants discussed recent experiences with nearby nature, for example, Participant 31 expressing a desire for scenes with an overview. She described an experience of vastness related to a scene containing many different elements:

*Participant 31: Recently we went to the train tracks, and I enjoyed that so much. We were standing next to the canal and a little train was passing us. We were standing on a dike and at the same time a huge boat entered using its horn. We were standing there and I said to the others: ‘guys, this is awesome! Watch the train, the boat and us with all the wheelchairs and everything. It is complete’. I enjoyed that so much.*

#### Desire for nostalgia

Some participants expressed the need for recognizable scenes, and the corresponding desire for nostalgia. Participant 11 argued that older adults in general, and people living with dementia in particular, could benefit from recognizable scenes.

*Participant 11: [I would like] a bit more variation and like I said, some recognizable things. Old people want to see things from the past.* […] *Especially for people, now I’m talking in general terms again, who are starting to suffer from dementia. Well, of course they live here a lot and they hang on to the past, they want some recognition. That's why I already said ‘things from the area here’. I think that adds something.*

Another participant discussed recognizable objects or elements in the nature scene, such as water mills, windmills or lighthouses.

*Participant 27: I think, for example, watermills and lighthouses are very beautiful.* […] *and I think that also appeals to people here, those Dutch elements.*

#### Desire for elements related to living situation

Some participants specifically expressed a preference for scenes from their own region (or from well-known areas from their past). Participant 37 for example expressed the wish to see scenes from the area where he currently lives (but cannot visit at the moment):

*Participant 37: You obviously have none [videos] of this area, do you? […] If you can see some of that again, well, I think that would be nice. […] I think there would be a lot of interest in that too, let me put it this way.*

Some participants indicated that they missed the area where they previously lived, pointing out different nature scenery. For example, participants 24 and 15 indicated that they missed the region of Friesland in the northern part of the Netherlands with lots of water, e.g., blue space, which provides possibilities for related activities such as water sports.

*Participant 15: Well, Friesland always attracts me. That's where you come from and it still pulls.*

*Participant 24: And I miss the water a bit, because I'm from Friesland, right? There are all kinds of lakes there. [If I had the chance] I would go back to Friesland, because I miss the water here so much.*

#### Desire for scenes associated with previous vacations

Landscapes that were associated with or resembled scenes participants knew from trips or vacations were mentioned often. Participant 24 talked about the vacations they used to enjoy together as a family. This illustrates the need for nostalgia as well as the need for recognition.

*Participant 24: Well, what I really like are lakes with boats and such. We used to go on holiday when the children were small, we always went to France, to a river, or to a lake or to the sea. Because our boys also loved water.*

Hence, in addition to generating a large variety of nature scenes that resemble nearby nature, scenes from popular vacation areas could be added to a large database of nature scenes as most participants recalled positive memories of vacations in the past, and indicated that they would enjoy seeing scenes that resembled those areas they remembered.

### Summary qualitative results

Findings of the semistructured interviews with older adults indicate that preferences regarding digital nature are highly person-related and connected to one's personal history. When participants would use a projection room in their daily life, most of them expressed the desire to experience different scenes every time. The scenes they would prefer to see vary widely, however. Some themes kept recurring, such as the distinction between nature nearby versus from all over the world, nature from one's personal living environment (either at present or in the past), nature that is associated with vacations in the past, nature with nostalgic elements and variety on a time scale such as seasonal changes. Throughout the interviews, the preference for blue spaces and scenes with a vantage point (providing an overview) was often mentioned.

To sum up, in order to keep users engaged when using the projection room in their daily lives, a large database with varying scenes is necessary.

## General discussion

In this study, a mixed-method approach was used to study the effects of digital nature videos on the social well-being of older adults (aged 62–99). The study entailed a survey and a semistructured interview. Quantitative results show an increase in connectedness (to the community) and related feelings of serenity, contentment and peacefulness after watching a digital nature video of 4 min. Qualitative findings further suggest a general preference for tended nature and for scenes providing overview, and they provide additional insights into how nature scenery could link to, among others, previous vacations and seasonal changes.

### Design implications

The qualitative results clearly indicate that older adults are an extremely heterogeneous group. Striving for a one-size-fits-all approach therefore is not feasible or realistic. The variety in answers to the question of what participants would like to experience in a projection room with digital nature suggests that a product/service should include a large database of scenes to select from, not only as a means to safeguard a variety of scenery in general, but also to address fluctuating needs throughout the day.

Nostalgia was an overall recurrent theme within the interviews when discussing desired additional digital nature content. This is in line with research showing that especially family-related nature experiences as a child determine the desire for nature experiences later in life.^45^ Considering the preference for variety across and within persons, we advise to ensure opportunities to adapt digital nature content to individual needs or mood states. To accommodate this, a large database with a large variety of scenes should be developed in future iterations.

Although no quantitative evidence was found for the preference for spacious nature, throughout the interviews, the preference for vastness and overview, sometimes in relation to blue space, was a recurring theme. Since exposure to blue space can have positive effects on general^46^ and psychosocial well-being,^47^ this is a promising direction for future digital nature development. These preferences are furthermore in line with literature in awe research stating that vast nature in general is associated with high levels of (perceived) connectedness with others and the world at large, and that it stimulates prosocial behavior.^19,34^

Considering that preferences for spacious (vs dense scenery) vary widely across older adults, incorporating transitions from spacious to dense scenery and vice versa seems advisable. Interestingly, this is in line with a suggestion by Appleton^48^ who, in a revision of his prospect-refuge theory, points out the emotional impact of contrasting experiences “involving the successive experiences of exposure to strongly contrasting landscape types” (p. 102). Considering the potential of VR technology for staging such dynamic experiences, clearly follow-up studies further exploring such scenarios are called for.

Finally, a major design implication relates to the human factor in nature scenery, either embodied by people present in the scene or by signs or suggestions of human presence such as benches affording social interaction or the sounds of children playing, indicating that one is not alone. Related preferences for tended nature scenes voiced in the interview sessions are in line with research pointing out that older adults value nature characteristics related to mobility (well-maintained paths and possibilities to sit down) and do not necessarily prefer wild nature.^49,50^ Although users do not actually visit the scene in real life, and therefore experience no real physical discomfort, nature characteristics related to mobility are nonetheless valued and when absent, this is marked as a negative point.

### Limitations and future research

As a result of the COVID restrictions and related lockdowns, participant recruitment amongst relatively frail older adults was difficult and, as a result, sample size was too low for quantitative analyses. Clearly, follow-up studies should include a larger population. Additionally, follow-up research should point out whether the (qualitative) findings of the present study are generalizable to the larger population of older adults.

Although not finding differences across the nature conditions might relate to sample size, this finding partly also aligns with previous research among older adults which likewise did not show a difference between spacious and dense nature scenery.^24^ Although not confirmed by our quantitative findings, a preference for tended scenery *did* transpire from our qualitative findings, mirroring findings from an aforementioned study in which social aspirations were markedly higher for tended digital nature walkthrough videos.^27^ Likewise, preferences articulated by some of our interviewees for scenes providing an overview (i.e., spacious rather than dense scenery) and preferences for vast blue spaces in general also warrant follow-up investigation.

Additionally, future research could explore the effects of different nature simulations under conditions where immersion is higher (e.g., using large projections or head-mounted displays). In line with these suggestions, the delivery mode of digital nature (i.e., the technology through which participants experience the scene), and whether or not one can navigate through the environment have been shown to be important with respect to enjoyment and well-being.^26^ Within the current study, we were limited to showing an immersive 3D video on a computer screen. Arguably, the length of the videos was relatively short (4–5 min) and immersion might be higher under longer viewing times. At the same time, previous research indicates that even brief exposure to nature and video content can already have positive effects on mood and well-being measures.^13,14,51^

Additionally, the recruitment of participants was biased towards older adults who were up for a social activity. As a result, we might have missed out on severely isolated older adults. Nonetheless, when considering the mobility of our participant group, we managed to include frail older adults with physical limitations who experience difficulties with going outside alone. Clearly, for these older adults in particular, digital nature can be considered a complementary strategy that allows them to experience (some of) the benefits of nature interaction.

## Conclusions

The findings of the present research demonstrate a significant increase in social connectedness scores and enhanced peacefulness after interacting with digital nature for frail older adults, as such validating the feasibility of using digital nature to improve social well-being among this heterogeneous group. Although digital nature should not be considered a replacement for outdoor nature interaction, our results testify to the potential of using digital nature as a complimentary strategy when interactions with outdoor nature become increasingly scarce and difficult due to aging. As such, our findings also warrant design approaches that seek to optimize digital nature scenery based on research findings, including the initial evidence-based design directions presented in the current research.

## Notes

1. Source video files: Dense tended nature—spacious tended nature: https://vimeo.com/402730560. Spacious tended nature—dense tended nature: https://vimeo.com/402730696. Dense wild nature—spacious wild nature: https://vimeo.com/402730817. Spacious wild nature—dense wild nature: https://vimeo.com/402730945.
